# Differential Mobility-Mass Spectrometry Double Spike Isotope Dilution Study of Release of β-Methylaminoalanine and Proteinogenic Amino Acids during Biological Sample Hydrolysis

**DOI:** 10.1038/s41598-017-18392-w

**Published:** 2018-01-08

**Authors:** Daniel G. Beach, Elliott S. Kerrin, Sabrina D. Giddings, Michael A. Quilliam, Pearse McCarron

**Affiliations:** Measurement Science and Standards, National Research Council Canada, 1411 Oxford St., Halifax, NS B3H 3Z1 Canada

## Abstract

The non-protein amino acid β-methylamino-L-alanine (BMAA) has been linked to neurodegenerative disease and reported throughout the environment. Proposed mechanisms of bioaccumulation, trophic transfer and chronic toxicity of BMAA rely on the hypothesis of protein misincorporation. Poorly selective methods for BMAA analysis have led to controversy. Here, a recently reported highly selective method for BMAA quantitation using hydrophilic interaction liquid chromatography-differential mobility spectrometry-tandem mass spectrometry (HILIC-DMS-MS/MS) is expanded to include proteinogenic amino acids from hydrolyzed biological samples. For BMAA quantitation, we present a double spiking isotope dilution approach using D_3_-BMAA and ^13^C^15^N_2_-BMAA. These methods were applied to study release of BMAA during acid hydrolysis under a variety of conditions, revealing that the majority of BMAA can be extracted along with only a small proportion of protein. A time course hydrolysis of BMAA from mussel tissue was carried out to assess the recovery of BMAA during sample preparation. The majority of BMAA measured by typical methods was released before a significant proportion of protein was hydrolyzed. Little change was observed in protein hydrolysis beyond typical hydrolysis times but the concentration of BMAA increased linearly. These findings demonstrate protein misincorporation is not the predominant form of BMAA in cycad and shellfish.

## Introduction

The non-protein amino acid β-methylamino-L-alanine (BMAA) has been implicated in the aetiology of neurodegenerative diseases, particularly amyotrophic lateral sclerosis/Parkinson’s disease complex (ALS/PCD) in humans^1^. BMAA was first identified in the 1960s as a natural product of the cycad plant (*Cycas micronesica*) during the search for the cause of ALS/PCD on the Pacific island of Guam^2^. The possibility that BMAA was the cause of ALS/PCD on Guam was later revived by hypotheses that BMAA could be biomagnified through its misincorporation into protein^3^ and that BMAA was a natural product of cyanobacteria growing symbiotically in the roots of the cycad^4^. It was also reported that BMAA production by cyanobacteria was not limited to the symbiotic *Nostoc sp*. in cycad roots but that it was produced in high levels by nearly all species of cyanobacteria investigated^5^. The validity of these findings^3–5^ have since been challenged by researchers in a number of fields including epidemiology^6^, cycad biology^7^ and analytical chemistry^8–10^.

Analysis of BMAA was originally carried out using reverse phase liquid chromatography with fluorescence detection (RPLC-FLD) for quantitation and single stage mass spectrometry (LC-MS) for confirmation of BMAA identity^3–5^. Derivatization chemistry was used to achieve retention of polar BMAA in RPLC and to attach a fluorescent tag enabling detection. This derivatization chemistry has broad specificity for all primary and secondary amino groups and has often been used in combination with single stage mass spectrometry for qualitative confirmation. Concentrations of BMAA as high as 1 g/kg in cyanobacteria^5^ and 3 g/kg in rodents^4^ have been reported using RPLC-FLD. Methods have since been developed using tandem mass spectrometry (MS/MS) detection in combination with either RPLC after derivatization^11–16^ or hydrophilic interaction liquid chromatography (HILIC) without derivatization^17–21^. The detection of g/kg levels of BMAA in biological samples has never been reproduced using these more selective methods, raising concerns about these original reports^8,9^. Results of BMAA quantitation using more selective MS/MS methods have been in good agreement in shellfish and diatoms with concentrations reported in the low- to sub- mg/kg. Even using MS/MS methods, inconsistencies in BMAA detection still exist for cyanobacteria and human tissue samples with some labs consistently reporting detection^11,22,23^ and others reporting negative results^9,18,24^.

Since the original report of higher recovery of BMAA from biological samples after strong acid hydrolysis^3^, sample preparation for BMAA analysis has largely been based on classical methods of strong acid protein hydrolysis. Recently, evidence has been published to suggest that the speciation of BMAA in biological samples is more complex than just “free” and “protein associated” fractions, with a large proportion being present in a third, “soluble bound” fraction^25,26^. Rosén *et al*. carried out a fractionation of low and high molecular weight compounds from neutral extracts of blue mussels and found that bound BMAA was predominantly present in the low molecular weight fraction^26^. Furthermore, the comparison of hydrolysis of these extracts using strong (6 M HCl) and mild (0.02 M HCl) acid showed that BMAA was released from its bound form under conditions not expected to lead to protein hydrolysis. The presence of a “soluble bound” fraction in cycad, shellfish and BMAA exposed zooplankton was simultaneously reported by an international collaborative study on BMAA analysis^25^. The fractionation of BMAA can therefore been defined as “free BMAA”, measured by extraction without strong acid hydrolysis, “total BMAA”, measured by total sample hydrolysis or “soluble bound BMAA”, measured by hydrolysis after sample extraction. The speciation of the “soluble bound” fraction remains unknown, and the reliable measurement of BMAA in biological samples remains a challenge.

Our group has recently reported two highly sensitive and selective new methods for quantitation of BMAA in biological samples, one using HILIC-differential mobility spectrometry-MS/MS (HILIC-DMS-MS/MS)^27^ and another using capillary electrophoresis-MS/MS (CE-MS/MS)^28^. Both these methods measure un-derivatized BMAA after total sample hydrolysis with limits of detection of 20 μg/kg dry sample. Compared with typical HILIC-MS/MS and RPLC-MS/MS methods used previously in our lab^17,18^, both HILIC-DMS-MS/MS and CE-MS/MS methods offer significant improvements in selectivity, limits of detection and confidence of positive identification. In particular, the higher separation resolution of hyphenated HILIC-DMS or CE eliminated interference from isomers and isobaric species, many of which are routinely observed in BMAA analysis^12,27^. Excellent quantitative performance has been achieved with both methods using isotope dilution calibration to compensate for matrix effects in ESI. Our work has consistently detected BMAA in shellfish and cycad, but all cyanobacteria samples we have analyzed to date have been negative for BMAA^17,18,27,28^.

DMS, with planar electrodes, or the similar high field asymmetric waveform ion mobility spectrometry (FAIMS) with concentric cylindrical electrodes, are relatively new gas phase ion mobility separation techniques. Applied to analytical mass spectrometry, DMS acts as an ion filter, separating ions produced by electrospray before they are detected in mass spectrometry. DMS and FAIMS have been used previously as primary separation tools for amino acid analysis by mass spectrometry, but not in combination with liquid chromatography. The separation of leucine and isoleucine, a typical benchmark for ion mobility spectrometry resolution of small molecules, was demonstrated early on in the development of FAIMS as a separation tool for analytical mass spectrometry at the National Research Council Canada^29^ and then again later using DMS^30^. Quantitation of the full suite of un-derivatized proteogenic amino acids in hydrolyzed biological samples has also been shown by ESI-FAIMS-MS/MS using flow injection analysis^31^.

Here, the recently reported HILIC-DMS-MS/MS method^27^ is expanded to include 17 proteogenic amino acids in order to make it useful in the investigation of the protein bound nature of BMAA in biological samples. We also developed a novel double spiking isotope dilution approach that allowed the study of BMAA extraction efficiency, hydrolytic release and stability in isolation from matrix effects in ESI. Through a variety of fractionations and hydrolysis experiments, these methods were applied to a detailed study of the extraction, fractionation and hydrolytic release of BMAA in shellfish and cycad.

## Results and Discussion

We recently reported the highly selective quantitation of BMAA in cycad and shellfish using HILIC-DMS-MS/MS^27^. First, two important modifications to this method were required to allow for a detailed study of the extraction, fractionation and hydrolytic release of BMAA. Two different isotopically labelled standards are now available for BMAA; D_3_-BMAA used in our original studies and ^13^C^15^N_2_-BMAA. It was desirable to develop a method that could selectively measure these two internal standards, each with an [M + H]^+^ precursor ion of *m/z* 122, using tandem mass spectrometry. Second, in order to optimize BMAA release and study its relationship to protein hydrolysis, a method suitable for analysis of BMAA and proteinogenic amino acids was desirable.

### BMAA Quantitation Using Double Spike Isotope Dilution

Several factors can lead to biases in BMAA quantitation including extraction recovery, BMAA stability under hydrolytic conditions and ESI signal suppression from the sample matrix. Fortunately, isotopically labeled standards for BMAA are now readily available and can be used to correct for one or all of these effects, depending on when during the protocol the labeled standard is spiked. A spike added to the initial sample corrects for most possible biases from sample preparation and analysis and is most often used in BMAA quantitation^11,19,25^, but this approach does not provide any information on the source of the bias. Furthermore, any instability of BMAA and internal standards under hydrolysis conditions would itself introduce a bias on quantitation and free and bound BMAA could be expected to exhibit differing stability under hydrolytic conditions.

**Figure 1:**
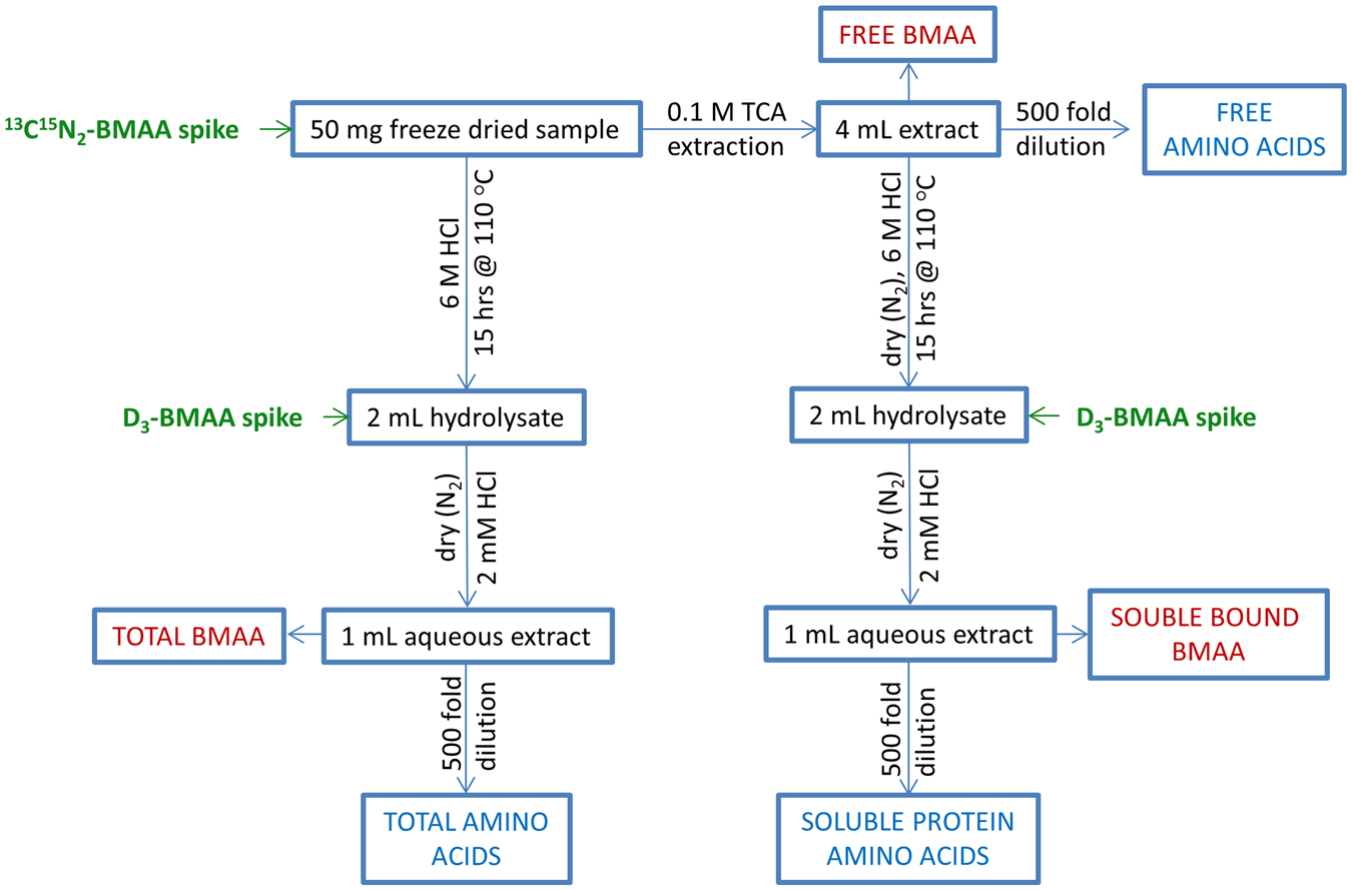
Summary of sample preparation workflows used in this study.

**Figure 2:**
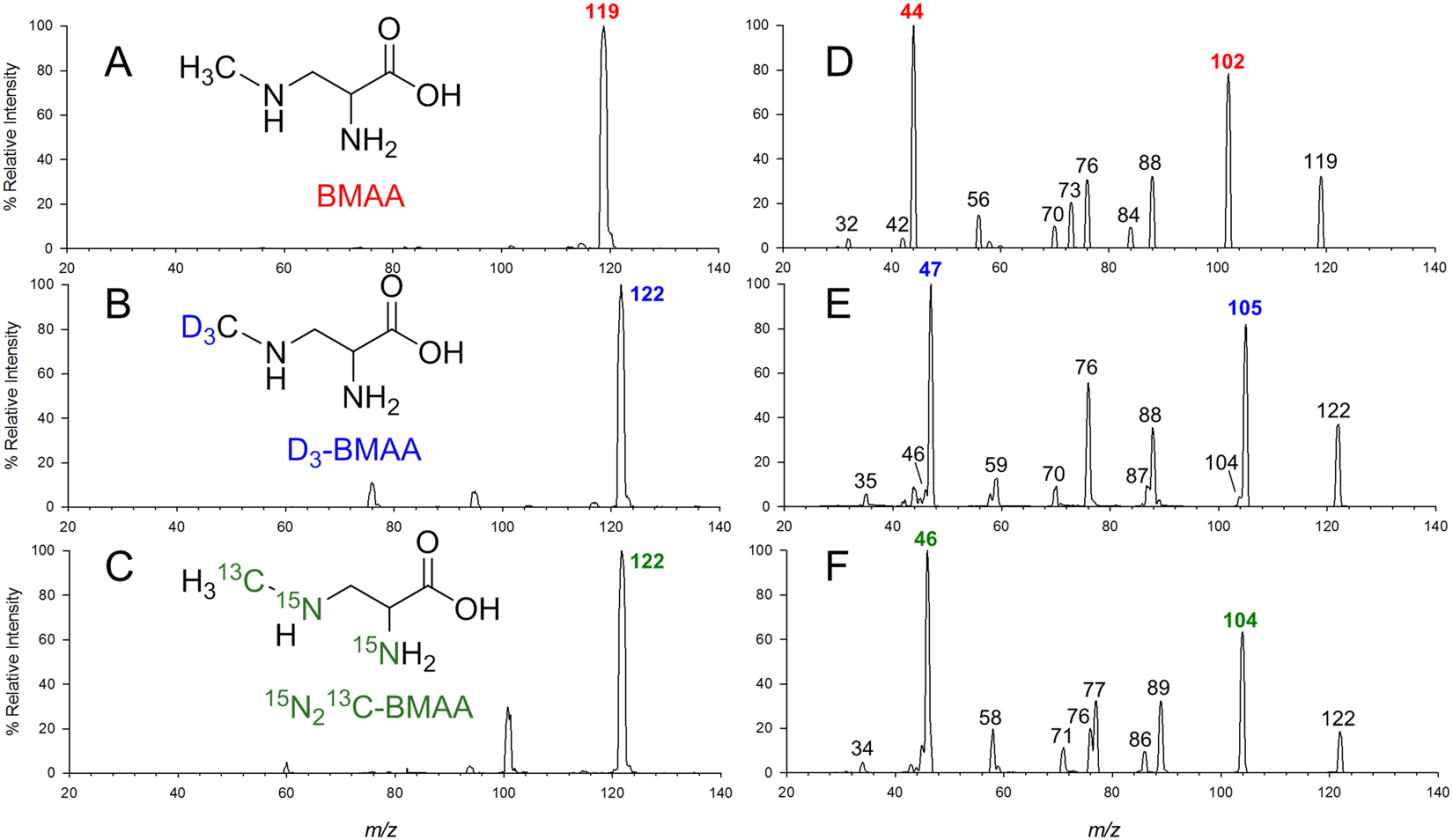
Full scan (**A**,**B**,**C**) and collision induced dissociation (**D**,**E**,**F**) spectra of protonated BMAA (**A**,**D**), D_3_-BMAA (**B**,**E**) and ^13^C^15^N_2_-BMAA (**C**,**F**). Product ions used in SRM experiment are shown in bold. Spectra were collected by infusion ESI-DMS-MS(/MS) using a collision energy spread of 15 V to 20 V.

A double spiking isotope dilution protocol was therefore developed to obtain information about both the stability and release of BMAA during hydrolysis, as well as correcting for matrix effects in ESI. Figure 1 shows a summary of the workflows used in this study, including the two spikes of labelled standard. ^13^C^15^N_2_-BMAA spiked prior to hydrolysis measured extraction recovery and BMAA degradation. D_3_-BMAA added after hydrolysis corrected for matrix effects in ESI and additional sample preparation steps such as filtration and allowed for quantitation of degradation of the initial standard spike. The challenge in developing this approach was to achieve selectivity between the two labelled standards, both with the same nominal *m/z* of 122. Figure 2 shows the full scan and product ion spectra of BMAA and its two isotopologues. The absence of electrospray background ions in these spectra is notable and is the result of using DMS for standard infusion experiments, as suggested previously^32^. From the data shown in Fig. 2, selective SRM conditions were developed to differentiate the three different isotopologues of BMAA, allowing them to be analyzed simultaneously. High resolution settings were required in Q3 to selectively differentiate product ions with adjacent *m/z* value such as ammonia loss from D_3_-BMAA at *m/z* 105 and from ^13^C^15^N_2_-BMAA at *m/z* 104. A flow injection analysis of the three isotopologues of BMAA was carried out to verify this selectivity and is shown in Fig. S1. This showed less than 0.5% interference arising from minor fragmentation pathways of 122 > 104 and 122 > 46 for ^13^C^15^N_2_-BMAA that can be detected in D_3_-BMAA transitions.

**Figure 3:**
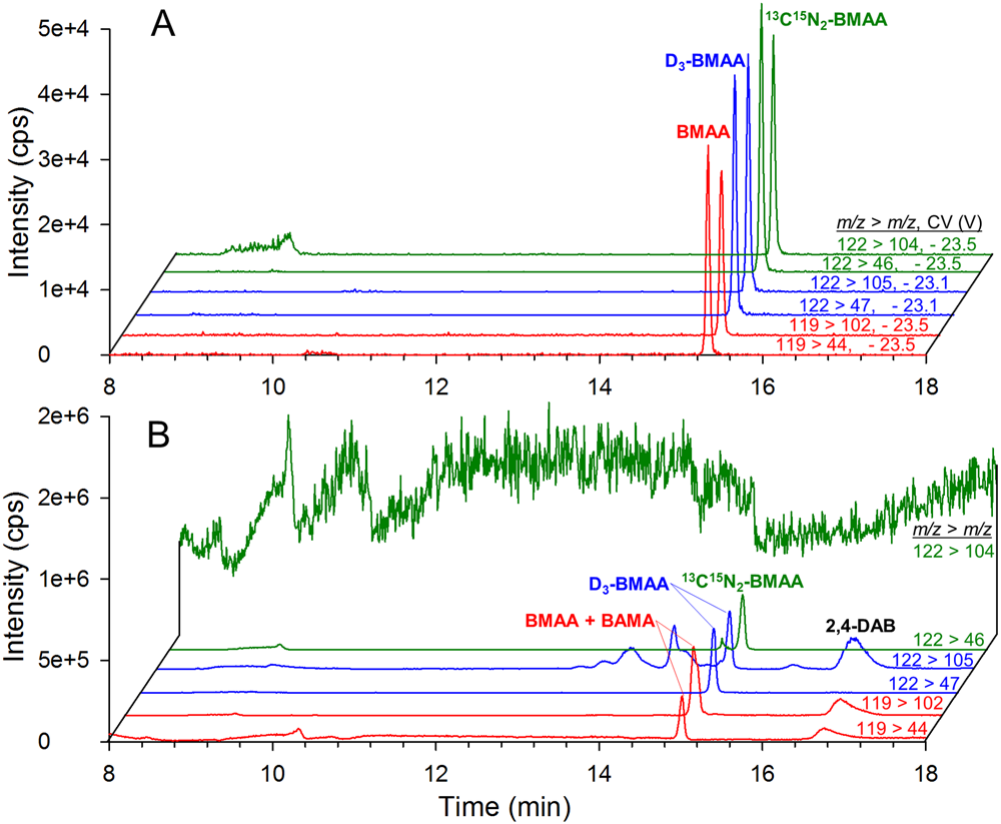
HILIC-DMS-MS/MS (**A**) and HILIC-MS/MS (**B**) chromatograms of naturally occurring BMAA, 125 nM spiked ^13^C^15^N_2_-BMAA and 125 nM spiked D_3_-BMAA in hydrolyzed mussel tissue RM.

Figure 3A shows HILIC-DMS-MS/MS chromatograms for a mussel tissue reference material (RM) spiked with 0.25 nmol ^13^C^15^N_2_-BMAA, hydrolyzed for 12 h at 100 °C, and then spiked with 0.25 nmol D_3_-BMAA. The use of DMS offers a significant benefit in selectivity and limits of detection compared to analysis of the same mussel tissue RM sample using an equivalent HILIC-MS/MS method without DMS (Fig. 3B). The *m/z* 119 > 44 transition of BMAA, the *m/z* 122 > 104 transition of ^13^C^15^N_2_-BMAA and the *m/*z 122 > 105 transition of D_3_-BMAA all have a high background when DMS is not used. This high background, that can be observed in general in ESI-MS analysis at low *m/z* values, can be attributed to chemical background from ESI and consists of a poorly characterized mixture of cluster ions, source fragments and electrochemical products of mobile phase components and system contaminants. The broader peak observed for BMAA in the *m/z* 119 > 102 transition in Fig. 3B is the result of an unresolved naturally occurring isomer of BMAA, β-amino-N-methylalanine (BAMA), interfering with BMAA detection. Using DMS, BAMA and BMAA are completely resolved and can each be selectively analyzed in the presence of a large excess of the other^33^. Without DMS, the more selective but less sensitive SRM transition *m/z* 119 > *m/z* 76 for BMAA can be used^19^. In addition to improved selectivity of BMAA analysis from isomers and chemical background, DMS also results in a signficant decrease in absolute ion counts detected, about 10-fold for BMAA in Fig. 3. Despide this decrease in absolute sensitivity, a significant increase in signal-to-noise ratio is observed and results in a corresponding decrease in limits of detection^27^.

### Amino Acid Analysis by HILIC-DMS-MS/MS

**Figure 4:**
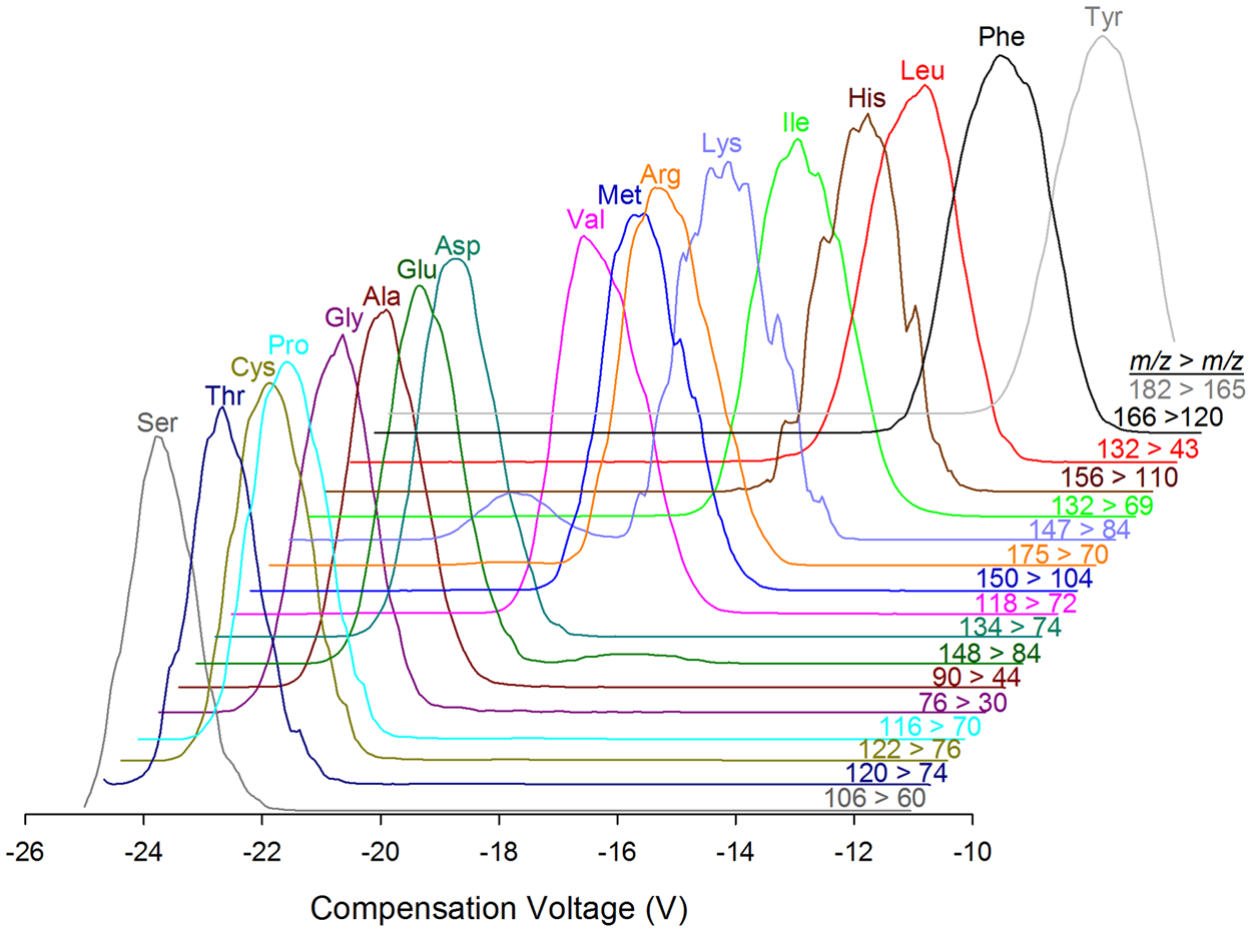
Separation of 17 proteinogenic amino acids in a mixed standard solution by ESI-DMS-MS/MS.

The first step to incorporating proteinogenic amino acids into our existing HILIC-DMS-MS/MS method for BMAA was to optimize the separation of amino acid standards by DMS. Recent work has shown that low concentrations (<1%) of acetonitrile are broadly suitable for separation of small polar analytes by FAIMS and DMS, including BMAA and its isomers^27,33–35^. Carrier gas modifiers in DMS help to amplify differences in mobility by inducing clustering of ions with neutral solvent molecules, thereby increasing selectivity of the separation. Without modifier, little separation is observed in DMS between small polar analytes such as amino acids. Using a modified carrier gas modifier solvent delivery system^27^ allowed us to fully explore the effect of varying low concentrations of acetonitrile in the carrier gas on amino acid separation, along with the effect of varying dispersion voltage (DV) (Fig. S2). Analyte dependant minima in compensation voltage of transmission were observed for most amino acids between 0.2% and 0.4% acetonitrile (Fig. S2A). A general trend of increasing resolution of amino acids with increasing DV was observed, but both acetonitrile concentration and dispersion voltage had an impact on sensitivity of analysis as well. Beyond 0.35% acetonitrile or 2600 V, many amino acids exhibited a significant decrease in sensitivity of analysis. These conditions, 0.35% acetonitrile and DV = 2600 V, correspond to those previously identified as optimum for separation of BMAA isomers^27^, and it was possible to analyze proteinogenic amino acids under the same DMS conditions as BMAA. Figure 4 shows the separation of 17 proteinogenic amino acids in a mixed standard by DMS-MS/MS using the SRM parameters in Table 1.

**Table 1:** HILIC-DMS-MS/MS parameters for analysis of BMAA and proteogenic amino acids.

Analyte	Retention Time (min)	Compensation Voltage (V)	Precursor ion *m/z*	Product ion *m/z*	Collision Energy (V)
BMAA	15.7	−23.5	119	102	13
				44	25
^13^C^15^N_2_-BMAA	15.7	−23.5	122	105	13
				47	25
D_3_-BMAA	15.7	−23.1	122	104	13
				46	25
Phenylalanine	6.3	−14.4	166	120	17
Leucine	6.4	−15.5	132	43	33
Isoleucine	6.6	−16.6	132	69	21
Methionine	7.2	−18.3	150	104	15
Valine	7.5	−18.6	118	72	17
Tyrosine	7.8	−13.0	182	165	14
Proline	7.9	−22.3	116	70	23
Alanine	9.1	−21.4	90	44	18
Glutamic acid	9.9	−21.0	148	84	20
Glycine	10.0	−21.9	76	30	20
Cysteine	10.1	−22.7	122	76	14
Threonine	10.1	−22.9	120	74	15
Aspartic acid	10.1	−20.9	134	74	19
Serine	11.1	−23.5	106	60	16
Histidine	17.4	−15.8	156	110	20
Arginine	17.7	−18.0	175	70	30
Lysine	18.1	−17.6	147	84	23

The datasets generated during the current study are available from the corresponding author on reasonable request.

**Figure 5:**
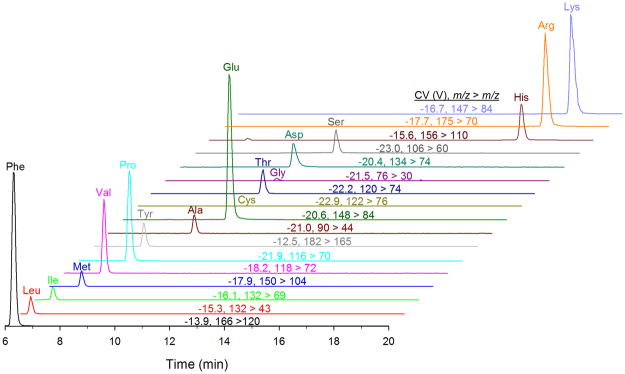
HILIC-DMS-MS/MS analysis of proteinogenic amino acids in a 500 fold dilution of a mussel tissue RM hydrolyzed for 12 h at 100 °C. All plots are normalized to the intensity of phenylalanine.

The established DMS-MS/MS parameters for amino acid analysis were combined with the existing HILIC gradient and DMS-MS/MS conditions for BMAA to develop a highly selective multidimensional HILIC-DMS-MS/MS method for BMAA and proteinogenic amino acids. This method is suitable for trace analysis of BMAA and amino acids, but for the intended application of measuring protein hydrolysis in biological samples, a concentration difference between of several orders of magnitude between trace BMAA and protein amino acids made it sub-optimal to measure both in a single run. This was primarily due to the poor HILIC peak shape and detector saturation observed for amino acids at these high concentrations. It was therefore desirable to analyze 500 fold dilutions of hydrolyzed samples in a separate injection for amino acid analysis, as shown in Fig. 5 for the hydrolyzed mussel tissue RM. Amino acid standards showed good linearity up 1 μM concentration and amino acids in diluted samples were estimated at an order of magnitude lower concentration. Though not required for the current application, the developed HILIC-DMS-MS/MS methodology would also be highly suited to the sensitive and selective trace analysis of proteinogenic amino acids in complex environmental or biological samples.

### Extraction and Fractionation of BMAA and Protein

The goal of the current work was to apply the developed HILIC-DMS-MS/MS methodology to study the hydrolytic release of BMAA and proteinogenic amino acids during strong acid hydrolysis of representative shellfish and cycad samples, while using a cyanobacterial RM negative for BMAA as a control sample. The developed methodology was suitable for measurement of the full suite of proteinogenic amino acids, but not all amino acids liberated from protein are stable to hydrolysis conditions. Based on previous reports of accurate, metrological protein quantitation by hydrolysis and amino acid analysis, proline, valine, phenylalanine, leucine and isoleucine were chosen as the most stable^36,37^. The relative peak area of these amino acids was compared across different sample preparation and hydrolysis procedures to give a relative measure of the amount of protein hydrolyzed.

**Figure 6:**
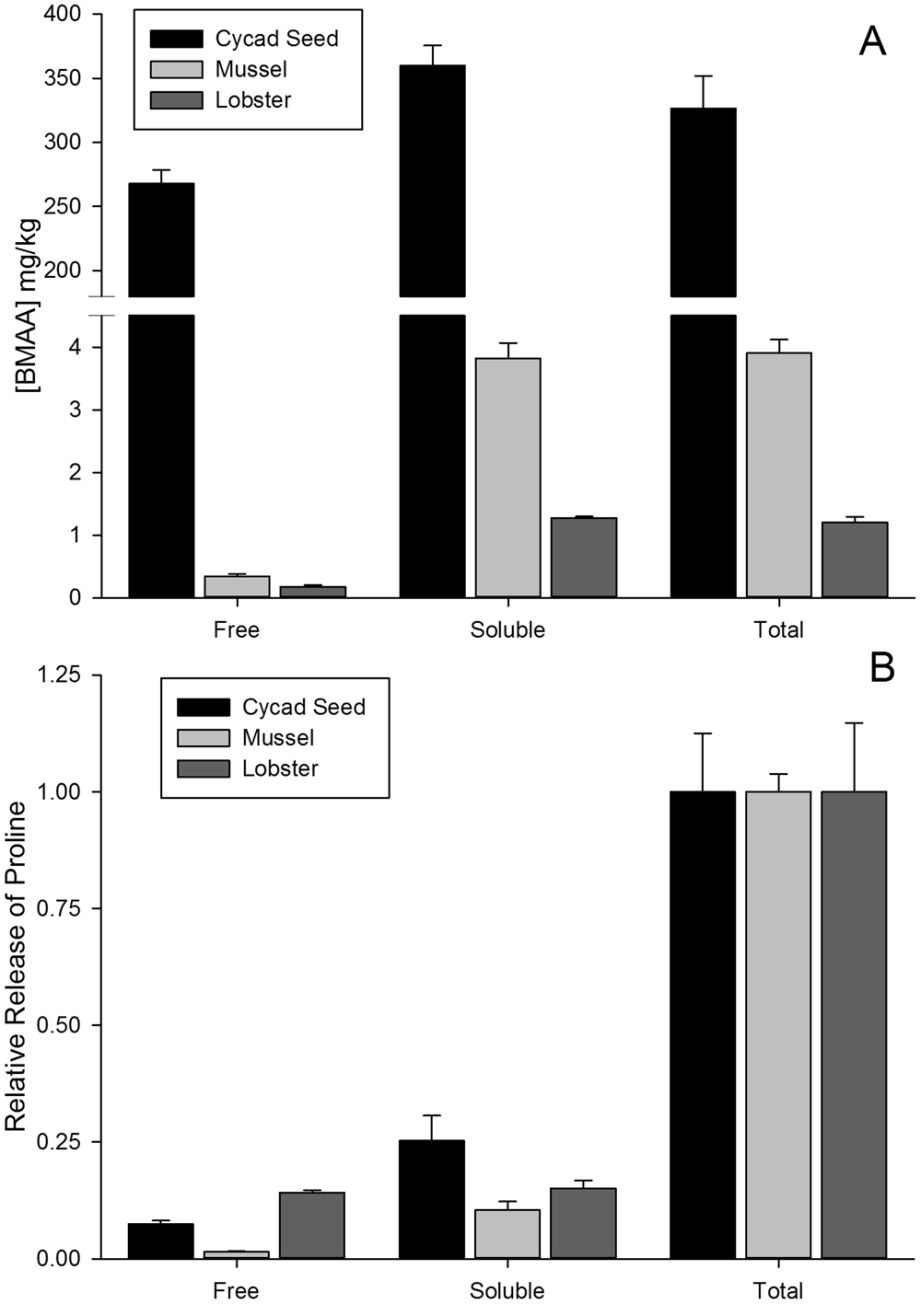
Distribution of BMAA (**A**) and protein as measured by hydrolytic release of proline (**B**) between free (unhydrolyzed), soluble (hydrolyzed) and total (hydrolyzed) cycad seed, mussel tissue RM and lobster samples. Error bars show standard deviations of triplicate sample preparations.

Three different sample preparation procedures were investigated: analysis of free BMAA, hydrolyzed soluble BMAA (soluble bound) and total sample hydrolysis without extraction, as summarized in Fig. 1. These were applied in triplicate to cycad and shellfish samples for analysis of BMAA and proteinogenic amino acids using the developed HILIC-DMS-MS/MS methodology (Fig. 6). Significant differences are observed between the distribution of BMAA in cycad and shellfish tissues. For both lobster and mussel, BMAA is predominantly present in the “soluble bound” fraction, with close to 10% present as free BMAA. For cycad seed, which contained much higher BMAA concentration than shellfish, about 75% of BMAA was present as free BMAA, but a significant amount of soluble bound BMAA was also detected. This result is consistent with recent reports of significant soluble bound fraction in mussels^25,26^, but showed a much lower proportion of soluble bound BMAA to free BMAA in cycad seed than reported previously^3,25^.

Figure 6 also demonstrates the significant difference between the extraction of protein and BMAA from all samples. In this respect, shellfish and cycad seed show a similar distribution, with the majority of protein remaining un-extracted in the pellet after TCA extraction. This result suggests that BMAA is not predominantly associated with protein in the extracts investigated. Instead, these results suggest the presence of some other unknown precursor to BMAA, which is released during hydrolysis. This notion is further supported by the observation that the majority of “soluble bound” BMAA in mussels is found in the low molecular weight fraction after filtration with a molecular weight cut-off filter (3000 Da), as reported previously^26^ and repeated here for shellfish and cycad (Fig. S3).

Hydrolysis procedures used in BMAA sample preparation have not been fully optimized for BMAA recovery, but are rather based on existing methods of protein hydrolysis. Considering BMAA appears not to be associated with protein in the samples investigated here, sample preparation conditions optimized for the hydrolysis of protein may not be optimal for BMAA analysis. A time course experiment was therefore carried out on the hydrolysis of the mussel tissue RM, with the goal of optimizing BMAA recovery from shellfish samples. Samples hydrolyzed for between 0.5 to 120 h were analyzed for BMAA and proteinogenic amino acids using the methods developed.

**Figure 7:**
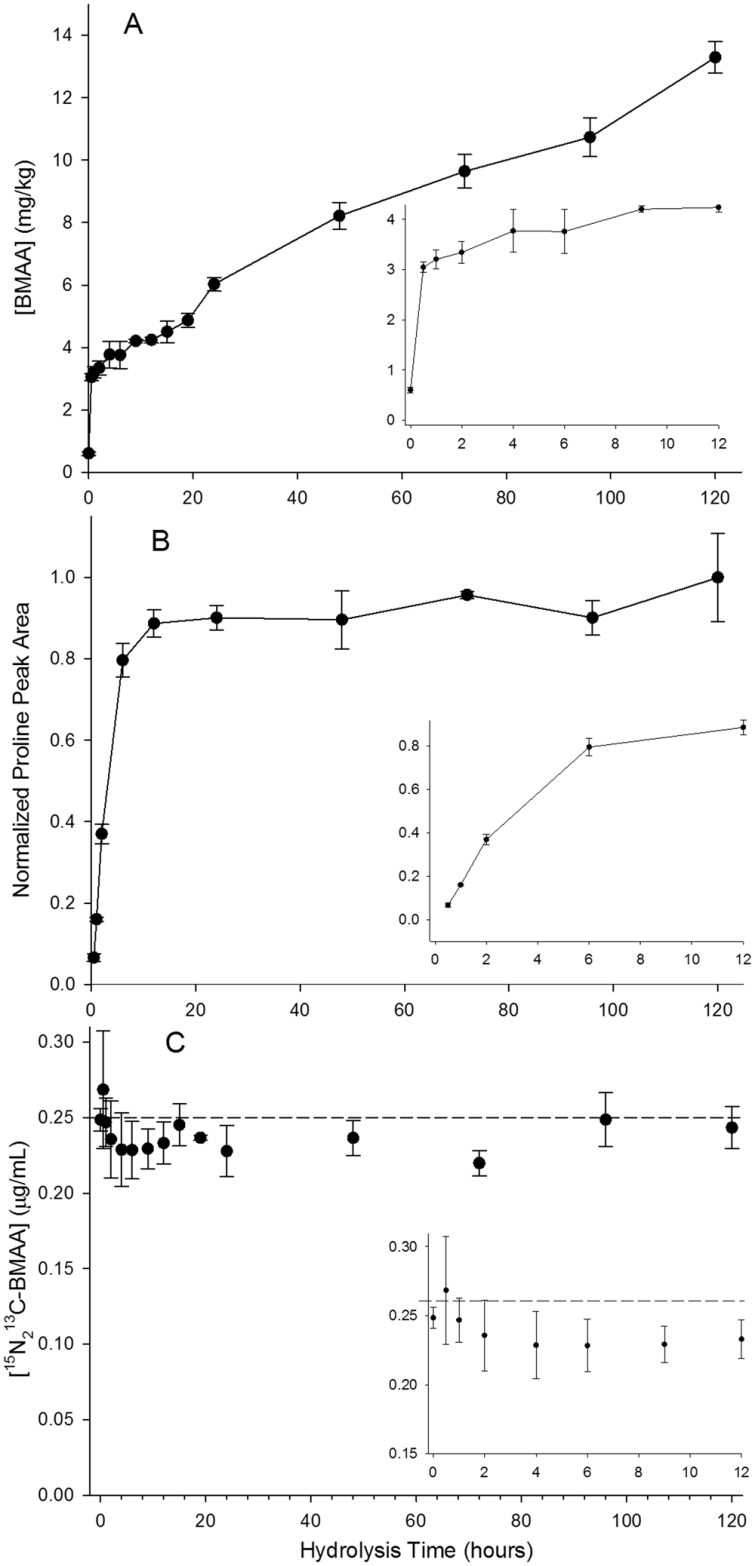
Release of BMAA (**A**), proline (**B**) and ^13^C^15^N_2_-BMAA internal standard stability (**C**) during strong acid hydrolysis of a mussel tissue RM. BMAA concentration determined by double isotope dilution using ^13^C^15^N_2_-BMAA spiked before hydrolysis. Error bars represent standard deviation of multiple sample preparations (2 ≤ N ≤ 6). Insets expand results from the first 12 h of the experiment.

Figure 7A shows the release of BMAA during the time course experiment. While hydrolysis times between 9–15 h gave BMAA concentrations of around 4 mg/kg, consistent with our previous analyses of this material, this represented only a fraction of the BMAA that was detected over longer hydrolysis times. In a series of increasingly longer experiments, our original 24 h experiment was extended to 120 h and BMAA levels continued to increase. Good reproducibility between up to 6 replicate samples prepared at each hydrolysis time was also observed. The BMAA concentrations shown in Fig. 7A are calculated using the ^13^C^15^N_2_-BMAA internal standards spiked into dry mussel tissue prior to hydrolysis. A constantly increasing concentration by this method of calibration would be expected in the case of internal standard degradation during hydrolysis . However, all samples were also spiked with D_3_-BMAA after hydrolysis and this spike was used to monitor the stability of ^13^C^15^N_2_-BMAA by directly measuring its concentration in each sample, as shown in Fig. 6C. This showed excellent stability of the internal standard over the 120 h time course experiment. The D_3_-BMAA spike added after hydrolysis was also used to monitor changes in ESI matrix effects throughout the time course experiment (Fig. S4). This showed a minor matrix effect of about 10% suppression in extractions of free BMAA from mussel tissue, but significant suppression of about 65% after protein hydrolysis was complete. This supports the previous suggestion that matrix effects observed in BMAA analysis in hydrolyzed samples by HILIC are predominantly due to high levels of proteinogenic amino acids^17^.

The developed methodology was also used to monitor the release of proteinogenic amino acids during the time course experiment. This showed an expected trend of increasing amino acid release for the first 12 to 48 h of hydrolysis, depending on the amino acid. Figure 7B shows the release of proline, which is stable under hydrolytic conditions. The four other stable amino acids, valine, leucine, isoleucine and phenylalanine gave similar results (Fig. S5). Two important differences can be observed between the release of BMAA (Fig. 7A) and that of proline (Fig. 7B). For the first 12 h of the time course experiment, BMAA is released much more quickly than protein is hydrolyzed. By about 18 h proline reaches a plateau, but beyond that BMAA continues to increase, with a nearly linear increase after 24 h for the remainder of the study period. This result was further confirmed by analysis of the same samples using a recently reported CE-MS/MS method^28^, which showed the same trend of increasing BMAA concentration.

Together, these results suggest two different compartments of bound BMAA in mussel tissue, the speciation of each differing significantly from non-specific protein incorporation. The rapid release of BMAA early in the hydrolysis is consistent with the previous suggestion of an unknown, low molecular weight conjugate that releases BMAA under mild hydrolysis conditions^26^. The continuous formation of additional BMAA at time points beyond protein hydrolysis may be unrelated and possible artefactual formation of BMAA during sample hydrolysis should be investigated. It is important to note however, that extended hydrolysis (48 hrs) of the control cyanobacterial RM did not lead to detection of BMAA.

## Conclusion

Here we have introduced new methodology that is highly suitable for studying the sample preparation and hydrolytic release of BMAA from biological samples. The double spiking isotope dilution approach for BMAA quantitation allowed BMAA stability during strong acid hydrolysis and ESI matrix effects to be studied independently. This will be of ongoing utility in BMAA method development where it is desirable to examine differences in recovery or stability in isolation from changing matrix effects in ESI. HILIC-DMS-MS/MS is also shown to be well suited to the selective analysis of protein amino acids and the hydrolytic release of stable amino acids is used to study the fractionation of protein and progress of hydrolysis. These methods could equally be applied to help elucidate the speciation of BMAA in other important biological samples such as microalgae and human tissue where BMAA has been reported previously.

The results presented clearly demonstrate that BMAA in shellfish and cycad seed is not broadly misincorporated into protein. It is instead present in some other form that appears to include at least two different sources or compartments. The rapid release of BMAA after only 0.5 h of hydrolysis suggests an unidentified, low molecular weight conjugate and is analogous to the recent observation that BMAA was released from mussels under milder acidic conditions^26^. Given that no maximum BMAA concentration from mussels could be observed, even after 5 days of strong acid hydrolysis, the possibility of artefactual formation of BMAA during the hydrolytic reaction should also be considered. It has also been suggested that BMAA could selectively bind to and be incorporated into other biopolymers, specifically the pigment melanin^38^. It is notable that melanin is also present in shellfish such as the mussels examined here and that the binding of polar neurotoxic paralytic shellfish toxins to melanin has also been previously shown^39^. However, the low aqueous solubility and high molecular weight of melanin make it an unlikely source of BMAA in the current study. Additional work is required for molecular identification of possible low molecular weight precursor to BMAA as well as to investigate the possibility of its artefactual formation during sample preparation.

## Methods

### Chemicals, Reagents and Samples

Optima LC-MS grade acetonitrile and a mixed L-amino acid standard (2.5 mM alanine, arginine, aspartic acid, glutamic acid, glycine, histidine, isoleucine, leucine, lysine hydrochloride, methionine, phenylalanine, proline, serine, threonine, tyrosine, valine and 1.2 mM L-cysteine in 0.1 M HCl) were purchased from Thermo Fisher Scientific (Mississauga, ON). Formic acid (>98% ACS grade) was purchased from Sigma Aldrich (Oakville, ON). β-N-Methylamino-L-alanine hydrochloride was obtained from Tocris Bioscience (Minneapolis, MN). D_3_-BMAA was initially synthesized in-house, as described previously^27^ and later purchased from Abraxis LLC (Warminster, PA). ^13^C^15^N_2_-BMAA dihydrochloride was purchased from Isoscience (King of Prussia, PA). The structure, purity and concentration of BMAA and internal standards was verified by ^1^H nuclear magnetic resonance spectroscopy (NMR)^40^. NMR stock solutions were diluted to 10 µM for BMAA and 25 µM for the internal standards and aliquoted into argon purged, flame sealed ampoules for use as stock solutions to prepare calibration standards.

Seeds of *Cycas thouarsii* were obtained from rarepalmseeds.com (Muenchen, Germany) and the endosperm of one seed was homogenized in a ball mill for 30 min, freeze-dried, and ground in a mortar and pestle. A steamed lobster (*Homarus americanus*) sample was purchased from a local supermarket (Halifax, Canada; May, 2016). Muscle tissue from the legs and tail were homogenized in a blender, freeze dried, and ground by mortar and pestle. A mussel (*Mytilus edulis*) tissue RM certified for several marine algal biotoxins (CRM-FDMT) was acquired from the National Research Council Canada (Halifax, Canada)^41–44^. A recently developed pilot scale cyanobacterial RM that has been extensively characterized for a wide range of cyanotoxins was used as a negative control for BMAA^45^.

### Sample Preparation

Two different methods were used for sample hydrolysis: A total tissue hydrolysis and a soluble fraction hydrolysis. The total tissue hydrolysis procedure was modified from our previous report^27^. Triplicate samples of 50 mg freeze-dried tissue were weighed into 5 mL amber glass ampoules with 2 mL 6 M HCl, spiked with 100 µL of 2.5 µM ^13^C^15^N_2_-BMAA and purged with argon before flame sealing. Hydrolysis was carried out in an oven at 110 °C for 15 h, unless otherwise specified. After hydrolysis, samples were spiked with 100 µL of 2.5 µM D_3_-BMAA, evaporated to dryness at 60 °C under nitrogen, reconstituted in 1 mL of 2 mM HCl, and filtered to 0.22 μm using a Millipore Ultrafree-MC spin filter. The soluble fraction hydrolysis procedure was adapted from Reveillon *et al* ^19^. Triplicate samples of 50 mg freeze-dried tissue were weighed into 5 mL reaction vials with 0.5 g of 75 μm acid washed glass beads (Sigma Aldrich, Oakville, ON) and 2 mL of 0.1 M trichloroacetic acid (TCA), spiked with 100 µL of 2.5 µM ^13^C^15^N_2_-BMAA and then shaken at 30 Hz for 30 min. Samples were centrifuged at 3100 × *g* and 15 °C for 10 min. Supernatant was transferred to a 5 mL amber ampoule and the extraction was repeated with an additional 2 mL of 0.1 M TCA. The pooled extract was either analyzed directly to measure free BMAA or was evaporated to dryness at 60 °C with a gentle stream of nitrogen and hydrolysed using the same conditions used in the total extraction prior to analysis. These sample preparation workflows are summarized graphically in Fig. 1.

For analysis of protein amino acids, a 500 fold sequential dilutions of sample hydrolysates in water were carried out using a Gilson Aspec GX 271 liquid handler (Mandel Scientific, Guelph, ON).

### HILIC-DMS-MS/MS Methods

LC separation was performed using a 5 μm TSKgel Amide-80 column (250 mm × 2 mm i.d.) (Tosoh, Grove City, OH, USA) held at 40 °C and a mobile phase of 50 mM formic acid in both water (A) or 95% acetonitrile (B) at a flow rate of 300 µL min^−1^. A linear gradient ran from 10 to 35% A over 15 min followed by a 6 min isocratic period, a linear gradient to 45% A over 1 min, an 8 min isocratic period, a 5 min column flush at 90% A and a 10 min equilibration at 10% A^17,27^.

Detection was carried out using an AB Sciex (Concord, ON, Canada) QTRAP 5500 mass spectrometer equipped with a Turbospray ionization source and SelexION differential mobility spectrometer. The DMS carrier gas modifier solvent delivery system was adapted, as described previously, to allow for delivery of a full range of modifier concentrations and better solvent evaporation and mixing^27^. Turbospray parameters included a spray voltage of + 5500 V, curtain gas of 20 psi N_2_, collision gas of 5 psi N_2_, desolvation gases of 45 psi (GS1) and 50 psi (GS2) N_2_, and a spray temperature of 400 °C. Mass spectrometer settings included a declustering potential of 140 V, an entrance potential of 10 V and exit potential of 13 V, DMS settings included separation (dispersion) voltage of 2600 V, a carrier gas modifier of 0.35% acetonitrile, a DMS temperature of 150 °C, and a DMS offset of −3 V.

DMS conditions for each amino acid were optimized by infusing the diluted amino acid standard at 10 µL min^−1^ into a 350 µL min^−1^ flow of 1:1 MeCN:H_2_O with 50 mM formic acid and collecting compensation voltage scans using selected reaction monitoring (SRM) with the transitions for each amino acid reported previously^46^. The concentration of acetonitrile in the N_2_ carrier gas was varied over a range of 0.04 to 2.5% and separation voltage was varied over a range from 2000 V to 4000 V. Optimized retention times, SRM transitions, collision energy, and DMS compensation voltages used for sample analysis are listed in Table 1.

## Electronic supplementary material

Supplementary information
