# Supplementary material for: Differential Mobility-Mass Spectrometry Double Spike Isotope Dilution Study of Release of β-Methylaminoalanine and Proteinogenic Amino Acids during Biological Sample Hydrolysis
Source: Sci Rep. 2018 Jan 8;8:117. doi: 10.1038/s41598-017-18392-w (PMC5758758; doi:10.1038/s41598-017-18392-w)
Supplement: Supplementary file 1 — Supplementary information [file 41598_2017_18392_MOESM1_ESM.pdf]

Supplementary Information

Differential Mobility-Mass Spectrometry Double Spike Isotope Dilution Study of Release of β-Methylaminoalanine and Proteinogenic Amino Acids during Biological Sample Hydrolysis

Daniel G. Beach*, Elliott Kerrin, Sabrina D. Giddings, Michael A. Quilliam, Pearse McCarron

Measurement Science and Standards, National Research Council Canada, 1411 Oxford St., Halifax, NS, B3H 3Z1, Canada

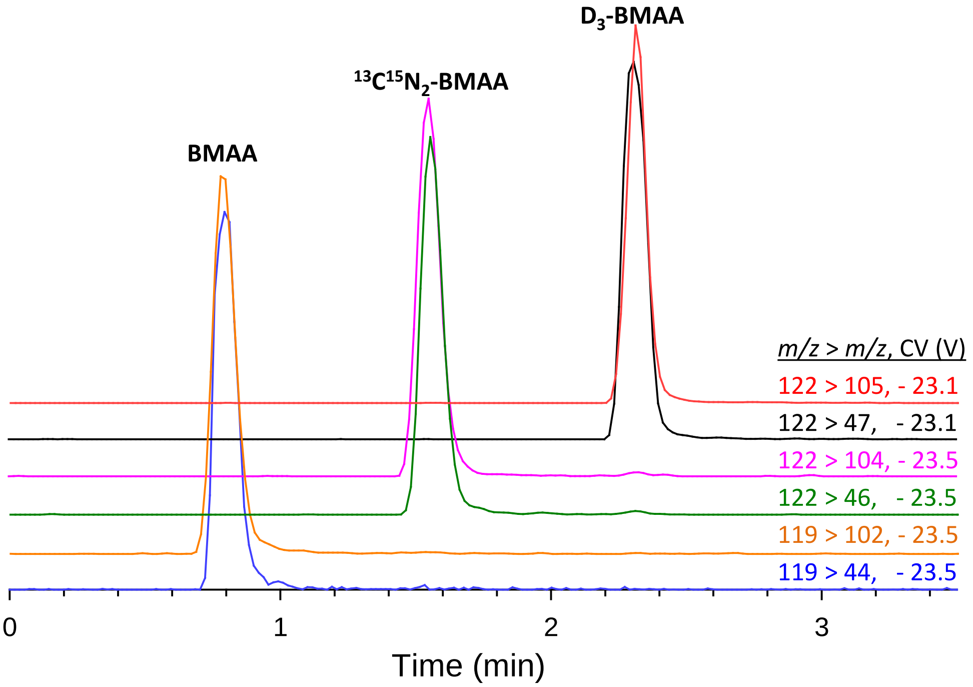

Figure S1: Selectivity of SRM method for resolving BMAA, D_3-BMAA and $^{13}C^{15}N_2$-BMAA assessed using flow injection analysis of a 5 µm solution of each standard.

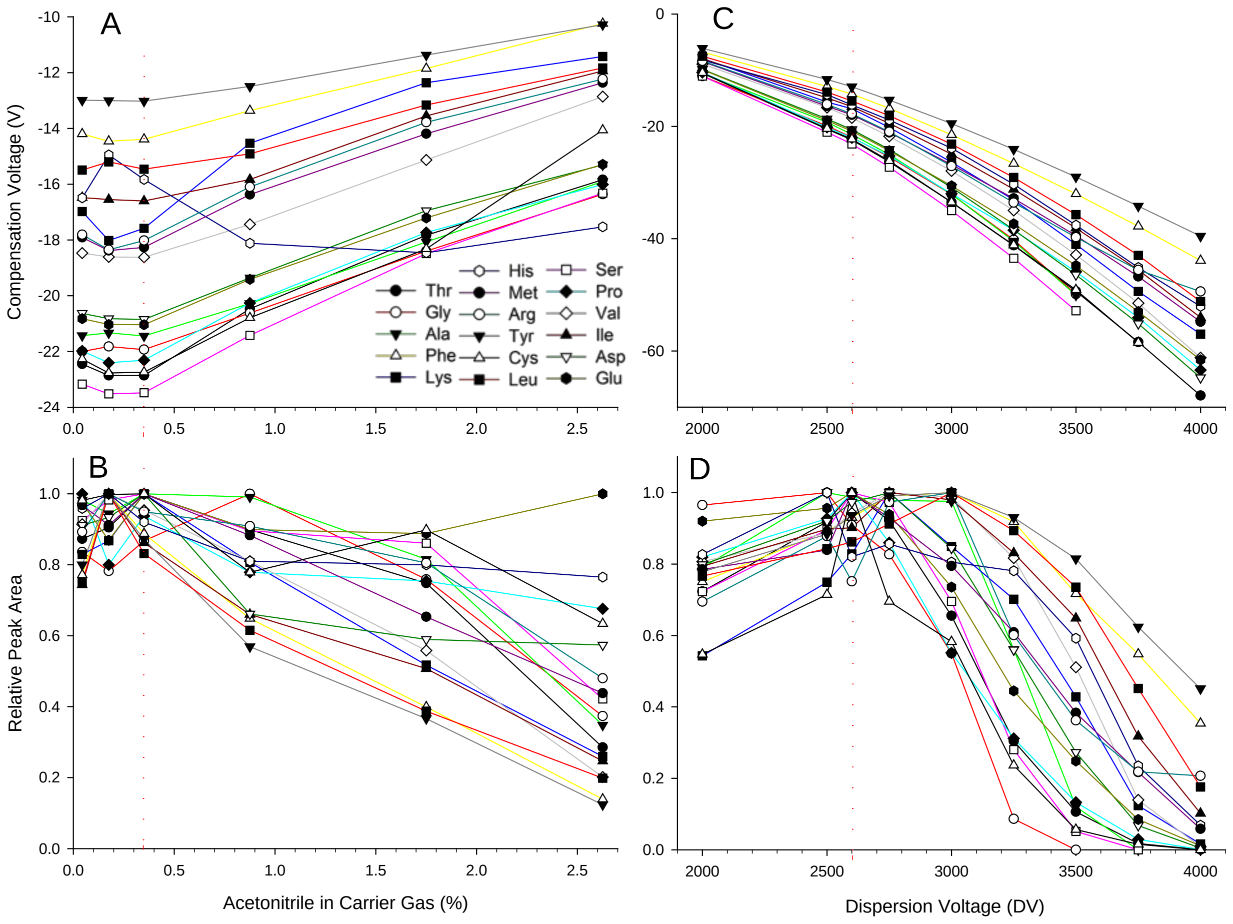

Figure S2: Impact of varying acetonitrile concentration in DMS carrier gas (A, B) or dispersion voltage (C,D) on separation (A, C) and sensitivity (B, D) of analysis of proteinogenic amino acids by ESI-DMS-MS/MS.

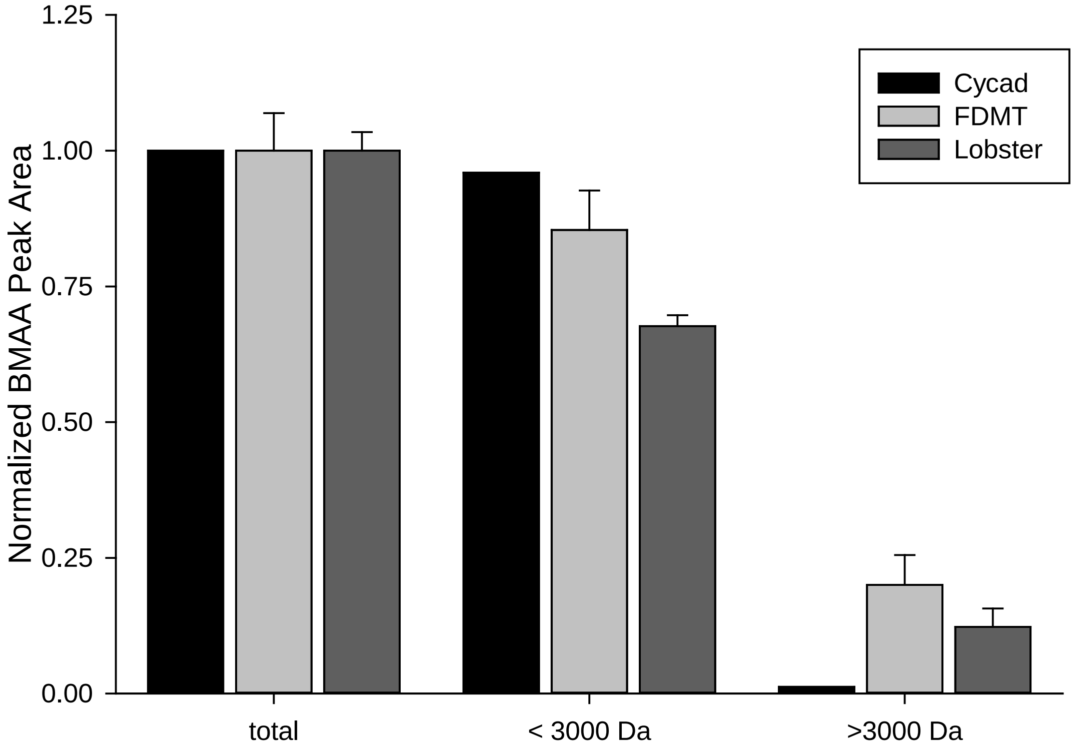

Figure S3: BMAA distribution between molecular weight cut-off fractions of cycad leaf (*Cycas debaoensis*), mussel tissue RM and lobster. Hydrolysis was carried out on TCA extracts and fractions were hydrolyzed to determine "soluble bound" BMAA. Error bars represent standard deviations of triplicate sample preparations.

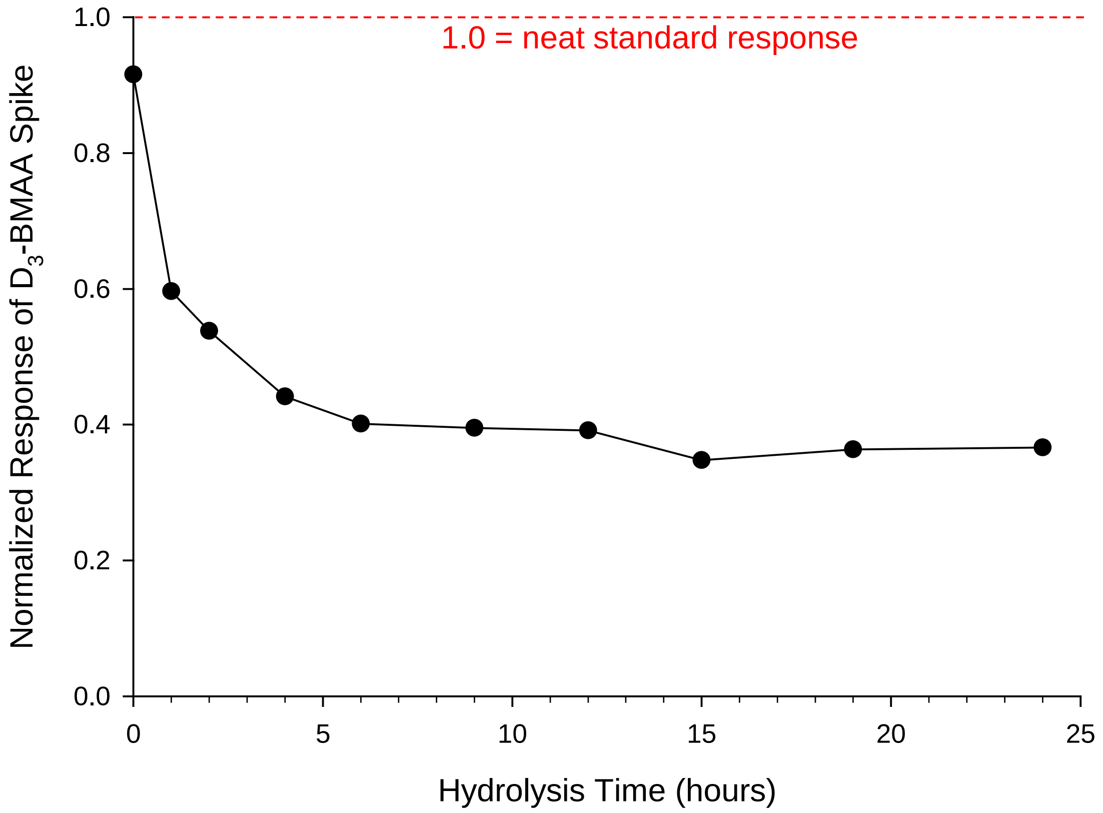

Figure S4: Increasing matrix effects in ESI with increasing hydrolysis time as measured by the sensitivity of the D_3-BMAA spike. Y-axis scale normalized to the response from a neat standard (no matrix effect).

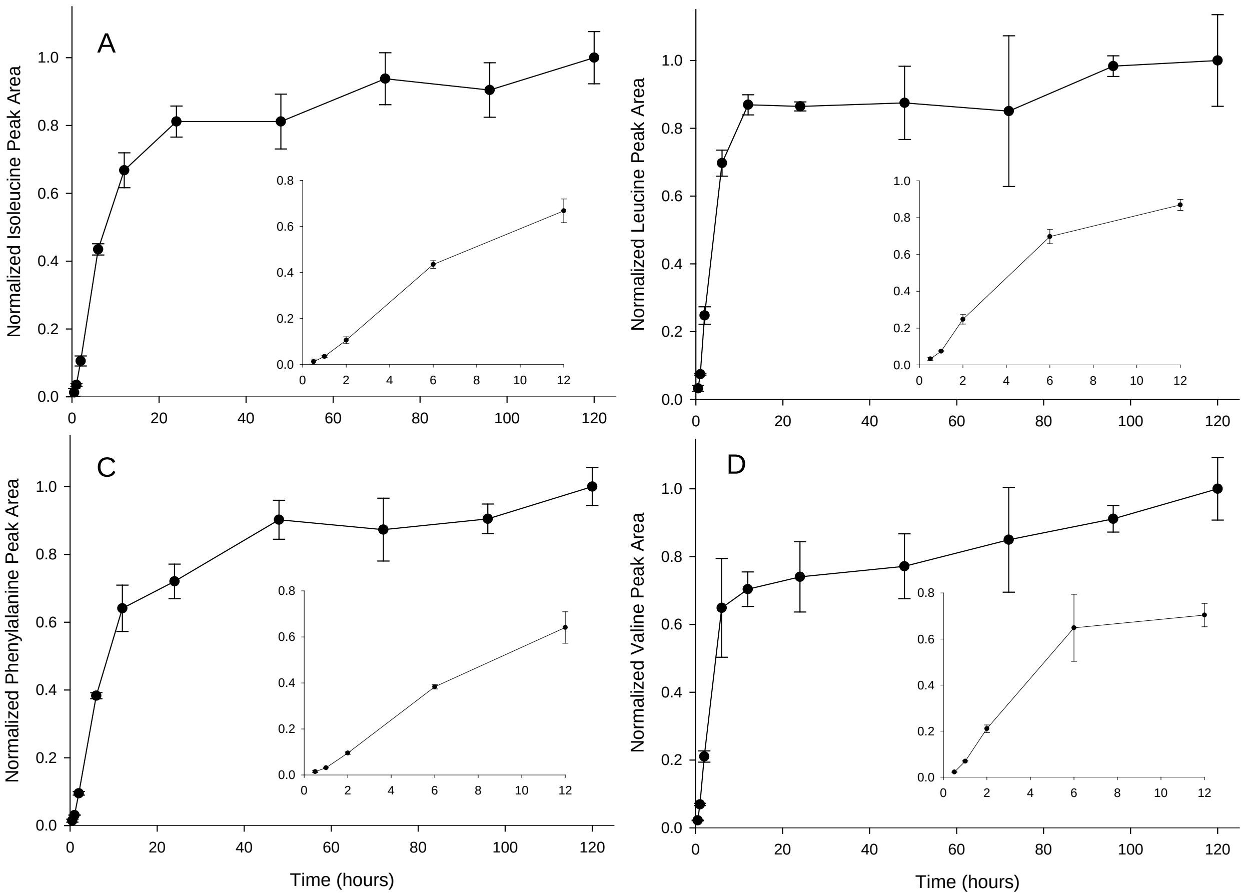

Figure S5: Release of stable amino acids isoleucine (A), leucine (B), phenylalanine (C) and valine (D) during strong acid hydrolysis of a mussel tissue RM. Error bars represent standard deviation of multiple sample preparations (N = 3). Insets show an expansion of the first 12 h of the experiment.
